# Non-thyroidal Illness Syndrome as an Adaptive Longevity Program: Reframing Low T3 in Acute and Chronic Disease

**DOI:** 10.7759/cureus.110397

**Published:** 2026-06-07

**Authors:** Angela D Mazza

**Affiliations:** 1 Endocrinology, Metabolic Center for Wellness, Oviedo, USA

**Keywords:** deiodinase, inflammation, low t3 syndrome, metabolic adaptation, mitochondrial function, non-thyroidal illness syndrome, reverse t3, thyroid hormone metabolism

## Abstract

Non-thyroidal illness syndrome (NTIS), historically termed euthyroid sick syndrome, is characterized by reduced serum triiodothyronine (T3), variable thyroxine (T4), and typically normal or suppressed thyroid-stimulating hormone (TSH) in the absence of intrinsic thyroid disease. Traditionally viewed as an adaptive response to acute illness that does not require intervention, NTIS is increasingly being recognized within broader contexts of metabolic adaptation, including aging, caloric restriction, and pharmacologically induced weight loss. This narrative review reexamines NTIS as a context-dependent metabolic reprogramming response that may represent an evolutionarily conserved survival and longevity mechanism. Evidence from critical care endocrinology, mitochondrial biology, aging research, caloric restriction studies, and emerging data on glucagon-like peptide-1 (GLP-1) receptor agonists is synthesized to explore the mechanistic and clinical implications of low T3 states. During acute physiologic stress, including infection, trauma, and starvation, reduced peripheral T4-to-T3 conversion and increased reverse T3 production appear to promote metabolic downshifting through decreased mitochondrial oxygen consumption, reduced anabolic signaling, and the redistribution of energy toward immune defense and cellular repair. These adaptations parallel pathways associated with enhanced metabolic efficiency and longevity. Similar thyroid hormone changes are increasingly observed in individuals undergoing significant weight loss, sustained caloric restriction, or GLP-1 receptor agonist therapy. While transient reductions in T3 may reflect adaptive energy conservation, persistent low T3 states in the setting of chronic inflammation, cardiometabolic disease, sarcopenia, or advanced aging may contribute to impaired mitochondrial function, reduced metabolic flexibility, and loss of physiologic resilience. NTIS may therefore represent a spectrum of adaptive and maladaptive responses influenced by physiologic context, duration, and inflammatory burden. A systems-based, longevity-oriented framework may improve the interpretation of low T3 states and help guide future research aimed at distinguishing beneficial metabolic adaptation from pathologic endocrine suppression.

## Introduction and background

Introduction: beyond the "do not treat" paradigm

Non-thyroidal illness syndrome (NTIS), historically referred to as euthyroid sick syndrome (ESS), describes a constellation of alterations in circulating thyroid hormone levels observed in patients with systemic illness in the absence of intrinsic thyroid disease. First characterized in hospitalized and critically ill populations in the 1970s, NTIS challenged the conventional assumption that abnormal thyroid function tests necessarily reflect primary thyroid pathology. Early investigations demonstrated that acute illness could profoundly alter peripheral thyroid hormone metabolism, independent of structural thyroid dysfunction [1-3].

The classic biochemical pattern of NTIS is characterized by reduced serum triiodothyronine (T3), elevated reverse T3 (rT3), and normal or low thyroid-stimulating hormone (TSH), with variable thyroxine (T4) concentrations depending on illness severity [1,4]. In mild or early illness, isolated reductions in T3 predominate ("low T3 syndrome"), whereas in more severe states, such as sepsis, trauma, or prolonged critical illness, both T3 and T4 may decline, often accompanied by suppressed TSH [2,5]. These alterations are largely attributed to changes in peripheral deiodinase activity, cytokine-mediated modulation of thyroid hormone metabolism, and hypothalamic-pituitary-thyroid (HPT) axis suppression [6,7].

For decades, NTIS has been widely interpreted as an adaptive, protective response to systemic stress. Reduced peripheral conversion of T4 to T3, along with increased production of metabolically inactive rT3, is thought to represent a coordinated metabolic downregulation that conserves energy during acute physiologic threat [4,8]. By lowering intracellular T3 availability, the organism reduces mitochondrial oxygen consumption, thermogenesis, and anabolic activity, prioritizing immune defense and cellular survival over growth and metabolic expenditure [9]. This framework has informed the prevailing clinical doctrine: abnormal thyroid function tests in the setting of systemic illness do not indicate thyroid failure and generally should not prompt thyroid hormone replacement [10].

While this paradigm remains appropriate in acute and critical care settings, its application to contemporary chronic disease warrants reassessment. The original NTIS literature emerged largely from intensive care unit (ICU) populations, sepsis cohorts, and acute trauma studies [1-3]. However, modern clinical practice increasingly encounters low T3 patterns in patients with chronic inflammatory conditions, cardiometabolic disease, advanced aging, prolonged caloric restriction, and pharmacologically induced weight loss. Emerging evidence suggests that persistent low T3 states in heart failure, chronic kidney disease, and systemic inflammatory disorders may correlate with adverse outcomes, including sarcopenia and increased mortality [11-13].

Moreover, advances in deiodinase biology, mitochondrial physiology, and immune-endocrine cross-talk reveal that thyroid hormone regulation is deeply integrated within broader metabolic and inflammatory signaling networks [6,7,9]. These insights challenge the simplicity of the traditional "do not treat" paradigm and raise a more nuanced question: under what conditions does thyroid hormone downregulation remain adaptive, and when might prolonged suppression contribute to impaired metabolic resilience?

From a clinical perspective, NTIS presents a recurring interpretive challenge. The characteristic laboratory pattern may raise concerns regarding thyroid dysfunction, yet in many cases reflects adaptive changes in hormone metabolism rather than primary thyroid disease. As low T3 states are increasingly recognized across diverse clinical settings, understanding the physiologic context underlying these findings has become increasingly important.

This narrative review integrates evidence from critical care endocrinology, mitochondrial biology, aging science, caloric restriction research, and contemporary metabolic medicine to examine the physiologic significance of low T3 states across diverse clinical contexts.

Accordingly, this review proposes that NTIS represents context-dependent metabolic reprogramming with distinct implications in acute versus chronic states. In acute illness, low T3 physiology may function as an evolutionarily conserved survival mechanism. In contrast, when sustained in the setting of chronic inflammation, aging, or prolonged energetic restriction, similar biochemical patterns may reflect persistent survival signaling with potential functional consequences. Reinterpreting low T3 states through a systems-based and longevity-oriented framework highlights the need to reconsider NTIS beyond the confines of critical care endocrinology, particularly in the context of aging, chronic inflammatory burden, and pharmacologically induced caloric modulation.

## Review

Mechanistic foundations of NTIS

Deiodinase Regulation and Peripheral Conversion

The hallmark biochemical alterations observed in NTIS arise primarily from changes in peripheral thyroid hormone metabolism rather than intrinsic thyroid gland dysfunction. Central to this process are the iodothyronine deiodinases, selenoproteins that regulate the tissue-specific activation and inactivation of thyroid hormones. Three deiodinase isoforms play distinct roles in thyroid hormone homeostasis: type 1 (D1), type 2 (D2), and type 3 (D3) [14,15].

D1, expressed predominantly in the liver, kidney, and thyroid, contributes to circulating T3 production through the outer-ring deiodination of T4. During acute illness, D1 activity declines, reducing peripheral T3 generation and contributing to rT3 accumulation [16]. D2, expressed in the brain, pituitary, skeletal muscle, and brown adipose tissue, regulates local intracellular T3 availability. Changes in D2 activity during systemic illness appear tissue-specific and may help preserve thyroid hormone signaling within critical organs such as the central nervous system [14,17].

In contrast, D3, an inactivating enzyme that converts T4 to rT3 and T3 to diiodothyronine (T2), is upregulated in inflammatory states and critical illness [16,18]. Increased D3 expression in the liver and skeletal muscle of critically ill patients supports the concept that NTIS reflects coordinated metabolic adaptation rather than primary thyroid failure [16]. Collectively, these deiodinase shifts lower systemic and tissue T3 availability while increasing rT3 production, resulting in the context-dependent modulation of thyroid hormone signaling.

Pro-inflammatory cytokines, particularly interleukin-6 (IL-6) and tumor necrosis factor-α (TNF-α), play a central role in mediating these enzymatic changes. Experimental models demonstrate that cytokine exposure suppresses D1 and D2 while inducing D3 expression, thereby shifting peripheral conversion toward T3 inactivation [19,20]. These cytokine-driven alterations provide a mechanistic bridge between systemic inflammation and thyroid hormone reprogramming.

Importantly, thyroid hormone signaling is highly tissue-dependent. Local deiodinase expression determines intracellular T3 concentrations independently of serum levels, underscoring the concept that NTIS reflects altered tissue thyroid economy rather than uniform systemic deficiency [14,15]. This distinction is critical when interpreting circulating hormone concentrations in the context of illness.

Inflammation and immune-endocrine cross-talk

NTIS occurs within the broader framework of the acute-phase response, a coordinated physiologic adaptation to infection, trauma, or systemic inflammation. Activation of innate immune pathways triggers cytokine release, hepatic acute-phase protein synthesis, and neuroendocrine modulation [21]. Thyroid hormone metabolism is tightly integrated into this response.

Nuclear factor kappa B (NF-κB), a central transcription factor in inflammatory signaling, has been implicated in the regulation of deiodinase expression and HPT axis suppression during illness [22]. Cytokine-mediated signaling at the level of the hypothalamus reduces thyrotropin-releasing hormone (TRH) expression, contributing to blunted TSH secretion in more severe or prolonged illness [23]. These central effects, combined with peripheral deiodinase shifts, reinforce the reduction in circulating T3.

The hypothalamic-pituitary-adrenal (HPA) axis also interacts dynamically with thyroid regulation during stress. Elevated cortisol levels, common in acute illness, may suppress TSH secretion and influence peripheral conversion pathways [24]. This coordinated endocrine response reflects a reprioritization of physiologic resources, favoring glucocorticoid-mediated stress adaptation over thyroid-driven metabolic activity.

From an evolutionary perspective, this immune-endocrine cross-talk appears designed to prioritize host defense and cellular survival. By reducing T3-dependent thermogenesis and anabolic signaling, the organism reallocates energetic resources toward immune activation and tissue repair [25]. Rather than representing endocrine failure, NTIS can therefore be understood as a structured metabolic shift embedded within the inflammatory response.

Mitochondrial energetics and metabolic flux

T3 is a principal regulator of mitochondrial function and systemic energy expenditure. T3 enhances the transcription of genes involved in oxidative phosphorylation, mitochondrial biogenesis, and substrate utilization [9]. It increases Na⁺/K⁺-ATPase activity, augments uncoupling protein expression, and stimulates oxygen consumption across multiple tissues [9]. Through these mechanisms, thyroid hormone governs basal metabolic rate and thermogenesis.

In the setting of acute illness, reduced T3 availability decreases mitochondrial oxygen consumption and ATP demand [9]. This downregulation may mitigate oxidative stress and limit reactive oxygen species (ROS) production during periods of systemic strain. Experimental models suggest that lowering thyroid-driven metabolic flux can preserve cellular integrity under hypoxic or inflammatory conditions [26]. Given the close interplay between oxidative stress and inflammatory signaling, it is also plausible that pathways such as nuclear factor erythroid 2-related factor 2 (Nrf2), a key regulator of cellular antioxidant defenses, may contribute to the adaptive metabolic responses observed in NTIS. However, the specific role of Nrf2 in NTIS remains incompletely characterized and warrants further investigation.

Such metabolic downshifting aligns with broader adaptive programs observed in starvation and critical illness, wherein reduced anabolic signaling and decreased energy expenditure enhance short-term survival [27]. By attenuating oxidative phosphorylation and limiting energy-intensive processes, the low T3 state may function as a protective brake on metabolic overactivation.

Collectively, alterations in deiodinase activity, inflammatory signaling, and mitochondrial energetics form a coherent physiologic logic: NTIS represents coordinated endocrine-metabolic reprogramming rather than random hormonal disturbance. Understanding these mechanisms provides the foundation for distinguishing adaptive acute responses from potentially maladaptive persistence in chronic disease and aging.

NTIS as an evolutionary survival program

The metabolic alterations observed in NTIS are not random endocrine perturbations but appear to reflect coordinated physiologic adaptation to systemic stress. Across diverse forms of acute threat, including infection, starvation, trauma, and critical illness, organisms demonstrate a conserved pattern of metabolic downregulation characterized by reduced energy expenditure, diminished anabolic signaling, and reprioritization of cellular resources toward survival [28,29]. Within this framework, the low T3 state of NTIS may represent an endocrine component of a broader survival program.

Thyroid hormone is a principal determinant of basal metabolic rate and thermogenesis. Reduced T3 availability during acute illness decreases thermogenic output, lowers oxygen consumption, and attenuates ATP-dependent processes [9]. This reduction in metabolic demand is particularly relevant during infection or trauma, when substrate availability may be limited and immune activation requires substantial energetic investment [29]. By suppressing energy-intensive processes such as protein synthesis, growth signaling, and thermogenesis, the organism reallocates metabolic resources toward immune defense and tissue repair.

Starvation and caloric deprivation provide additional insight into the adaptive nature of thyroid hormone downregulation. Prolonged fasting and caloric restriction consistently lead to reductions in circulating T3 and increases in rT3, paralleling the biochemical signature of NTIS [30,31]. These changes are associated with decreased resting energy expenditure beyond what would be predicted by weight loss alone, a phenomenon termed adaptive thermogenesis [32]. Rather than reflecting pathology, this endocrine shift enhances energetic efficiency during nutrient scarcity.

Experimental models of caloric restriction further demonstrate that reduced thyroid hormone signaling is intertwined with broader longevity-associated pathways. Caloric restriction is associated with decreased metabolic rate, modulation of mechanistic target of rapamycin (mTOR) signaling, reduced insulin/IGF-1 activity, and enhanced cellular stress resistance [27,33]. Reductions in energy availability are also accompanied by decreased leptin signaling, which may suppress hypothalamic TRH expression and contribute to downstream reductions in thyroid hormone production and signaling. In this context, leptin may function as an important integrator of nutritional status and neuroendocrine adaptation during energetic stress. Thyroid hormone interacts with many of these pathways. T3 stimulates mTOR activity and promotes anabolic processes, including protein synthesis and mitochondrial biogenesis [34]. Consequently, reduced T3 signaling during energetic stress may function in parallel with mTOR attenuation to suppress growth pathways and favor maintenance and repair.

Lower metabolic flux has also been linked to reduced production of ROS and diminished oxidative damage [35,36]. By decreasing mitochondrial oxygen consumption, the low T3 state may mitigate oxidative stress during periods of systemic strain [27]. This effect aligns with longevity models in which mild metabolic suppression enhances cellular resilience and extends lifespan in multiple species [27,33].

Importantly, observations in human aging provide further context. Several studies have reported that lower-normal thyroid function, including modestly elevated TSH or reduced T3 levels within the reference range, is associated with increased longevity in older populations [13,37]. While causality remains uncertain, these findings suggest that mild attenuation of thyroid signaling may, under certain conditions, align with extended survival rather than endocrine failure.

Taken together, these lines of evidence support the hypothesis that NTIS, particularly in acute contexts, may represent an adaptive metabolic downshifting program embedded within conserved survival biology. Reduced T3 availability during systemic stress appears to conserve energy, limit oxidative burden, suppress anabolic signaling, and reprioritize physiologic resources toward immune competence and cellular repair. Framed in this light, acute NTIS may represent not dysfunction but a regulated endocrine expression of evolutionary resilience.

The modern context: GLP-1 therapy and intentional caloric modulation

The classic NTIS literature emerged primarily from acute and critical care settings. However, contemporary metabolic medicine increasingly encounters low T3 patterns in non-ICU populations undergoing intentional weight loss, prolonged caloric restriction, or pharmacologic appetite modulation. The rapid expansion of GLP-1 receptor agonist therapy has introduced a modern physiologic context in which energetic flux is deliberately altered. Understanding thyroid adaptation within this framework is essential for accurate clinical interpretation.

Caloric Restriction and Thyroid Adaptation

Sustained caloric restriction consistently induces reductions in circulating T3 concentrations in humans, even in the absence of systemic illness [30,31]. Both short-term fasting and long-term energy deficit reduce peripheral T4-to-T3 conversion while increasing rT3 levels [30]. These changes contribute to a measurable decline in resting energy expenditure beyond what is predicted by changes in body composition alone, a phenomenon termed adaptive thermogenesis [33].

Adaptive thermogenesis reflects a coordinated endocrine response designed to improve metabolic efficiency during energy scarcity [32]. Reductions in T3 lower mitochondrial oxygen consumption and ATP turnover, attenuating energy expenditure at the cellular level [9]. In weight loss studies, decreases in serum T3 correlate with reductions in resting metabolic rate and increased energetic efficiency [38]. Importantly, these hormonal adaptations occur in otherwise healthy individuals and are generally interpreted as physiologic responses rather than thyroid pathology.

Long-term caloric restriction trials in humans have similarly demonstrated modest but sustained reductions in T3 levels accompanied by favorable changes in cardiometabolic risk markers [39]. Such findings reinforce the concept that reduced thyroid hormone signaling can be part of a broader adaptive metabolic recalibration during negative energy balance.

Thus, the biochemical pattern characteristic of NTIS, particularly the isolated low T3 state, may arise in settings of intentional caloric modulation without representing intrinsic thyroid dysfunction. These observations provide an important physiologic bridge between classic illness-associated NTIS and modern metabolic interventions.

GLP-1 receptor agonists and energetic signaling

GLP-1 receptor agonists have transformed the management of obesity and type 2 diabetes by inducing significant appetite suppression, delayed gastric emptying, and sustained caloric deficit [40,41]. Rapid and substantial weight loss achieved with these agents results in predictable reductions in energy expenditure. Changes in thyroid hormone signaling may contribute to this adaptation, although the relative contribution of thyroid-mediated mechanisms remains incompletely defined [42].

Although large-scale trials have not demonstrated clinically significant thyroid hormone suppression in euthyroid individuals, emerging clinical observations suggest that some patients undergoing rapid weight reduction may exhibit reductions in serum T3 within the reference range or modestly below it [43]. When observed, these findings appear to correlate with the degree of caloric deficit and weight loss and may be consistent with adaptive thermogenesis rather than primary thyroid disease.

Mechanistically, GLP-1-induced negative energy balance activates many of the same pathways engaged during caloric restriction, including reduced insulin signaling, altered substrate utilization, and shifts in energy expenditure [40,41]. Given the known sensitivity of deiodinase activity to energetic status and inflammatory signaling, it is biologically plausible that GLP-1-associated metabolic shifts engage some of the adaptive thyroid pathways observed during fasting or illness. However, direct evidence linking GLP-1 therapy to NTIS-like physiology remains limited.

Importantly, this interpretation does not imply that GLP-1 receptor agonists induce pathologic thyroid dysfunction. Rather, when reductions in T3 are observed during substantial weight loss, they may reflect physiologic energy conservation responses embedded within human metabolic regulation. Distinguishing adaptive endocrine recalibration from intrinsic thyroid pathology requires careful attention to clinical context, including baseline thyroid status, degree of weight loss, inflammatory burden, and symptom trajectory.

In this modern therapeutic landscape, low T3 states may emerge not only from acute illness but also from intentional energetic modulation. Recognizing these parallels supports the hypothesis that aspects of NTIS physiology may represent a form of context-dependent metabolic reprogramming. Whether activated by infection, starvation, trauma, or pharmacologically induced caloric deficit, several common adaptive features, including reduced thyroid hormone signaling and altered energy expenditure, may reflect shared responses to energetic stress. Further research is needed to determine the extent to which these physiologic states represent overlapping or distinct adaptive programs.

While emerging evidence suggests potential mechanistic overlap between NTIS, caloric restriction, and GLP-1-induced metabolic adaptation, prospective studies are needed to clarify the clinical significance of low T3 states in these settings and to distinguish adaptive endocrine responses from pathologic thyroid dysfunction.

Acute vs. chronic NTIS: when adaptation becomes dysregulation

While the metabolic alterations characteristic of NTIS appear adaptive in the setting of acute physiologic stress, increasing evidence suggests that persistent low T3 states in chronic disease may carry distinct physiologic implications. Distinguishing between transient endocrine adaptation and prolonged metabolic suppression is therefore essential for contextualizing NTIS within modern clinical medicine.

Acute NTIS

In acute illness, including sepsis, trauma, major surgery, and critical care states, NTIS is widely regarded as a protective metabolic adaptation [2,5]. The rapid reduction in circulating T3 concentrations observed in these settings occurs within hours to days of physiologic stress and is accompanied by increased rT3 production and the suppression of HPT axis activity [8].

This endocrine shift reduces energy expenditure during periods when metabolic resources must be redirected toward immune defense and tissue repair. Lower T3 availability decreases mitochondrial oxygen consumption, thermogenesis, and anabolic activity, thereby limiting ATP demand during physiologic crisis [9]. Such metabolic reprioritization may enhance short-term survival by conserving energy and mitigating oxidative stress in metabolically vulnerable tissues [26].

Clinical observations support the adaptive nature of these changes. In critically ill populations, the magnitude of T3 reduction often correlates with illness severity, suggesting that the endocrine response scales with physiologic stress [8]. Although low T3 levels have been associated with mortality in some cohorts, interventional trials of thyroid hormone replacement in acute NTIS have not demonstrated clear benefit, reinforcing the interpretation that these alterations reflect systemic adaptation rather than primary endocrine failure [44].

Consequently, most clinical guidelines advise against routine thyroid hormone treatment in critically ill patients with NTIS, emphasizing that abnormal thyroid function tests in the context of acute illness should generally be interpreted as secondary physiologic responses [10].

Chronic NTIS

In contrast to the transient hormonal shifts observed in acute illness, persistent low T3 states have increasingly been described in chronic inflammatory and cardiometabolic conditions. Patients with heart failure, chronic kidney disease, obesity-related metabolic dysfunction, and systemic inflammatory disorders frequently exhibit biochemical patterns consistent with low T3 syndrome outside the critical care setting [11,45].

Chronic low-grade inflammation appears to play a central role in sustaining these alterations. Cytokine-mediated suppression of deiodinase activity, combined with ongoing metabolic stress, may perpetuate reduced T3 availability even in the absence of acute physiologic threat [19]. Over time, this persistent endocrine profile may contribute to impaired mitochondrial efficiency, altered substrate utilization, and reduced metabolic flexibility.

Several studies have demonstrated that low T3 levels in chronic disease states correlate with adverse clinical outcomes. In patients with heart failure, reduced T3 concentrations are independently associated with increased mortality and disease severity [11]. Similar associations have been observed in chronic kidney disease, where low T3 levels correlate with inflammation, protein-energy wasting, and reduced survival [46].

Beyond organ-specific disease, persistent low T3 physiology may also intersect with age-related decline in physiologic resilience. Thyroid hormone plays a critical role in skeletal muscle metabolism, mitochondrial biogenesis, and protein turnover [9]. Sustained reductions in T3 availability may therefore contribute to sarcopenia, muscle weakness, and impaired functional capacity in older adults [47]. These changes may in turn increase vulnerability to frailty, metabolic inflexibility, and diminished recovery from physiologic stress.

Emerging evidence also suggests that post-viral inflammatory states, including post-acute sequelae of SARS-CoV-2 infection (commonly termed "long COVID"), may involve persistent alterations in thyroid hormone metabolism and energy regulation [48]. However, the available data remain limited, and it is currently unclear whether these findings represent classic NTIS physiology, post-viral metabolic adaptation, persistent inflammatory signaling, or a combination of these mechanisms. While the mechanisms remain incompletely understood, ongoing inflammatory signaling and mitochondrial dysfunction have been proposed as contributing factors.

Taken together, these observations suggest that the physiologic meaning of NTIS may depend largely on duration and context. In acute illness, transient reductions in T3 appear to function as a protective metabolic adaptation. In contrast, when similar hormonal patterns persist in the setting of chronic inflammation, cardiometabolic disease, or aging, the resulting metabolic suppression may contribute to impaired mitochondrial resilience, muscle catabolism, and reduced physiologic reserve.

This distinction highlights a central thesis of the present framework: the clinical significance of NTIS is not defined solely by hormone concentrations but by the broader metabolic environment in which they occur. Duration of endocrine alteration and cumulative inflammatory burden likely determine whether NTIS physiology remains adaptive or transitions toward maladaptive metabolic suppression.

Longevity implications

A growing body of research suggests that thyroid physiology may play a nuanced role in human aging and longevity. While much of this literature derives from observational aging studies rather than direct investigations of NTIS, it provides a useful framework for considering how reduced thyroid hormone signaling may intersect with mechanisms of metabolic adaptation and resilience. Overt hypothyroidism is clearly associated with adverse metabolic consequences; however, several observational studies have reported that individuals with exceptional longevity often demonstrate modestly reduced thyroid function compared with younger populations [13,36]. These findings have prompted renewed interest in the possibility that mild attenuation of thyroid signaling may, under specific conditions, align with mechanisms that promote metabolic efficiency and extended lifespan.

Studies of centenarian cohorts have consistently demonstrated subtle shifts in thyroid function parameters, including higher serum TSH concentrations and relatively lower circulating thyroid hormone levels within the normal range [36,48]. Genetic analyses have also identified polymorphisms associated with reduced thyroid hormone signaling that appear more prevalent among long-lived individuals [49]. While causality remains uncertain, these observations support the hypothesis that modest reductions in thyroid-driven metabolic activity may represent a physiologic phenotype compatible with extended survival.

One possible explanation for this association lies in the relationship between thyroid hormone signaling and systemic energy expenditure. Thyroid hormones are potent regulators of basal metabolic rate, mitochondrial activity, and thermogenesis [9]. Sustained reductions in thyroid signaling may therefore decrease metabolic flux, limiting oxidative stress and cumulative cellular damage over time [35]. Such mechanisms parallel broader longevity pathways observed in caloric restriction and other metabolic interventions that reduce energy turnover while enhancing cellular maintenance and repair processes [27,33].

However, this relationship introduces an important physiologic paradox. While reduced metabolic rate may confer long-term advantages related to oxidative stress and energy efficiency, excessive suppression of thyroid-driven metabolism can also impair essential physiologic functions. Thyroid hormone is critical for skeletal muscle maintenance, mitochondrial biogenesis, and neuromuscular performance [9,11]. In older adults, inadequate thyroid hormone signaling may therefore contribute to sarcopenia, reduced functional capacity, and increased frailty risk [50].

This tension highlights the delicate balance between metabolic efficiency and physiologic resilience. Aging organisms must simultaneously conserve energy and preserve functional capacity. When thyroid signaling is modestly attenuated in otherwise healthy individuals, the resulting metabolic efficiency may support longevity. Conversely, when reductions in thyroid hormone availability occur in the context of chronic inflammation, systemic disease, or advanced physiologic decline, similar biochemical patterns may contribute to progressive functional impairment.

**Figure 1:**
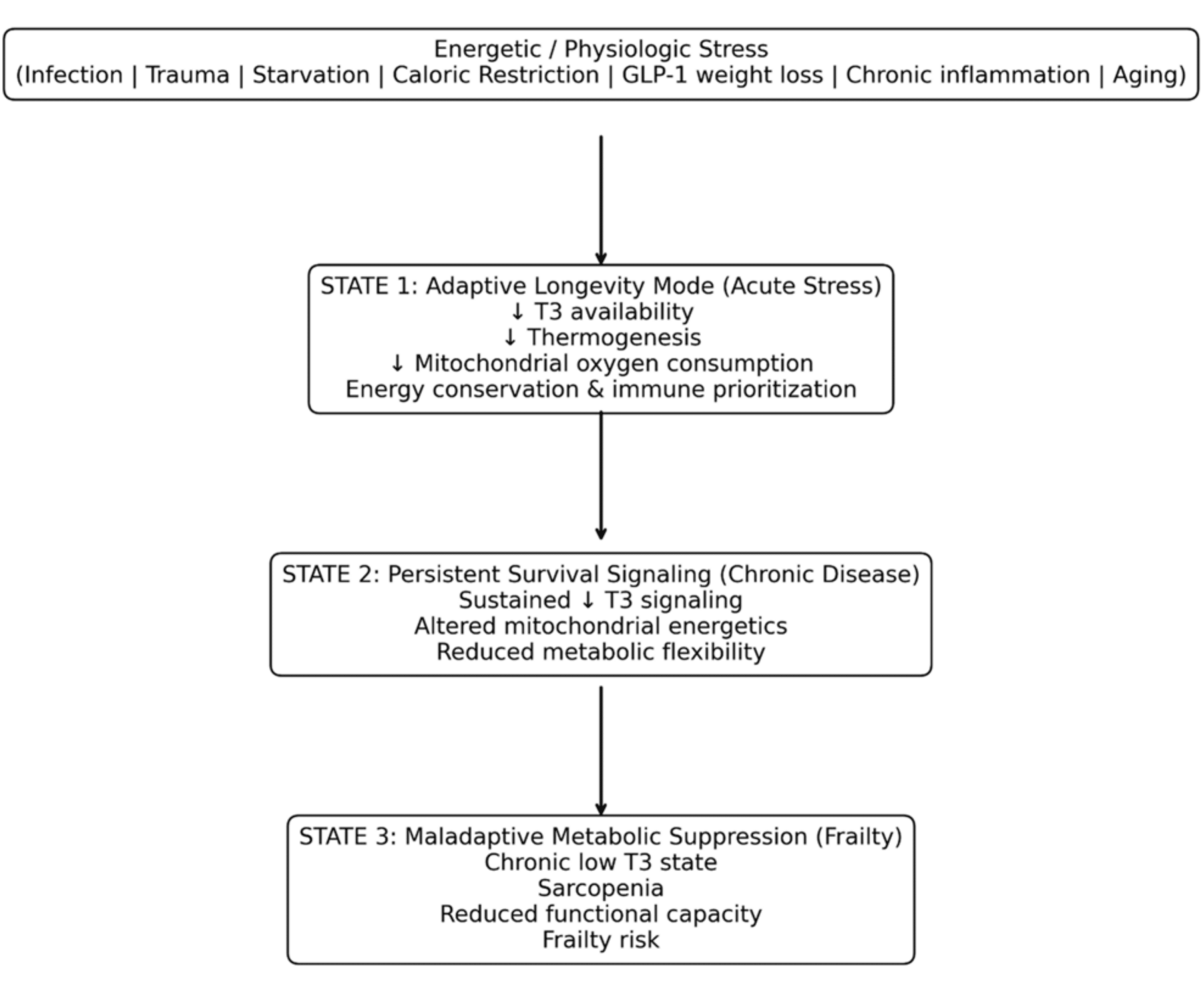
Continuum of thyroid adaptation in non-thyroidal illness syndrome GLP-1: glucagon-like peptide-1; T3: triiodothyronine Non-thyroidal illness syndrome may represent a dynamic metabolic continuum rather than a binary endocrine disorder. During acute physiologic stress, reduced T3 availability promotes energy conservation and cellular protection ("adaptive longevity mode") [2,5]. When systemic stress or inflammation persists, prolonged metabolic downshifting may lead to reduced physiologic reserve ("persistent survival signaling") [11,45]. In advanced aging or chronic disease, sustained suppression of thyroid-driven metabolic activity may contribute to sarcopenia, frailty, and impaired metabolic resilience ("maladaptive metabolic suppression") [50]. The physiologic meaning of low T3 states therefore depends on context, duration, and underlying systemic health. Image created by Angela D. Mazza using Microsoft PowerPoint (Microsoft Corporation, Redmond, Washington, United States)

Within this framework, NTIS can be conceptualized as part of a broader continuum of metabolic adaptation rather than a binary endocrine disorder. To conceptualize this continuum, a three-state model of thyroid-mediated metabolic regulation is proposed (Figure 1).

State 1: Adaptive Longevity Mode (Acute Stress)

During acute physiologic stress, such as infection, trauma, or short-term caloric deprivation, transient reductions in T3 availability promote energy conservation, reduce oxidative burden, and redirect metabolic resources toward immune defense and cellular repair. In this context, low T3 physiology represents an adaptive survival mechanism embedded within conserved evolutionary biology.

State 2: Persistent Survival Signaling (Chronic Inflammation)

In chronic inflammatory or cardiometabolic conditions, sustained activation of stress-responsive pathways may maintain suppressed thyroid hormone signaling beyond the acute phase. While initially adaptive, persistent metabolic downshifting may gradually impair mitochondrial efficiency, reduce anabolic capacity, and limit physiologic reserve.

State 3: Maladaptive Metabolic Suppression (Frailty)

When prolonged reductions in thyroid-driven metabolic activity occur alongside aging-related decline in physiologic resilience, the resulting endocrine profile may contribute to sarcopenia, reduced mobility, metabolic inflexibility, and increased vulnerability to stressors. In this stage, metabolic suppression may shift from adaptive conservation to functional limitation.

Importantly, these states are not defined solely by thyroid hormone concentrations but by the broader metabolic and inflammatory environment in which they occur. The same biochemical pattern, particularly reduced circulating T3, may therefore carry distinct physiologic implications depending on context, duration, and underlying systemic health.

Recognizing this continuum provides a useful framework for interpreting low T3 states within longevity medicine. Rather than viewing NTIS solely as a transient laboratory abnormality or as a pathologic endocrine deficiency, it may be more appropriately understood as a dynamic metabolic signaling state that reflects the organism's current energetic priorities. From this perspective, the clinical challenge lies not in correcting laboratory values, but in understanding when metabolic downshifting remains protective and when it may signal declining physiologic resilience.

Clinical interpretation in precision endocrinology

Interpretation of thyroid function tests in the context of NTIS requires careful clinical judgment. The biochemical alterations characteristic of NTIS, particularly reduced circulating T3, have traditionally been interpreted through a binary framework: either dismissed as benign laboratory artifacts of illness or viewed as potential indicators of thyroid hormone deficiency. However, emerging insights into thyroid hormone metabolism, inflammatory signaling, and systemic energy regulation suggest that such simplified interpretations may not adequately capture the physiologic complexity underlying these hormonal changes.

Current clinical guidelines appropriately caution against routine thyroid hormone replacement in patients with NTIS, particularly in the setting of acute illness [10]. In critically ill populations, thyroid hormone alterations typically reflect systemic metabolic adaptation rather than intrinsic thyroid gland dysfunction. Interventional trials evaluating thyroid hormone therapy in these settings have not demonstrated consistent benefit, reinforcing the importance of avoiding reflexive pharmacologic intervention based solely on laboratory values [44].

At the same time, the increasing recognition of persistent low T3 states in chronic disease highlights the limitations of reflexively dismissing these findings without broader clinical evaluation. Low T3 levels have been associated with adverse outcomes in a range of cardiometabolic and inflammatory conditions, including heart failure, chronic kidney disease, and systemic inflammatory disorders [11,45]. In such contexts, thyroid hormone alterations may serve less as isolated endocrine abnormalities and more as biomarkers of underlying metabolic stress or physiologic vulnerability.

For clinicians practicing precision endocrinology, interpretation of NTIS therefore requires contextual assessment rather than automatic therapeutic or diagnostic responses. Several key factors may help clarify the physiologic meaning of low T3 states.

First, the presence of acute systemic illness remains the most common and well-established cause of transient NTIS. In hospitalized or critically ill patients, reduced T3 concentrations generally reflect short-term metabolic adaptation and should not be interpreted as evidence of primary hypothyroidism [2,5].

Second, clinicians should consider energetic flux, including active weight loss or sustained caloric restriction. As discussed previously, reductions in circulating T3 commonly accompany negative energy balance and adaptive thermogenesis during weight loss [32,38]. In such settings, thyroid hormone changes may represent physiologic energy conservation rather than endocrine dysfunction.

Third, evaluation of chronic inflammatory burden is essential. Persistent low-grade inflammation, common in cardiometabolic disease, autoimmune disorders, and aging, can alter deiodinase activity and thyroid hormone signaling [19]. When low T3 patterns occur in conjunction with inflammatory markers or chronic disease states, they may reflect ongoing metabolic stress rather than transient adaptation.

Fourth, clinicians should assess indicators of musculoskeletal health and physiologic reserve, including sarcopenia, reduced physical performance, or frailty. Thyroid hormone plays a critical role in skeletal muscle metabolism and mitochondrial function, and prolonged reductions in T3 availability may contribute to diminished functional capacity in vulnerable populations [47,50,51].

Taken together, these considerations support a shift from binary interpretation toward a more nuanced, context-based framework. This aligns with a systems-based interpretation of thyroid physiology, in which mitochondrial function, hormonal signaling, and inflammatory tone are interdependent rather than isolated variables. Rather than asking whether NTIS should be treated or ignored, clinicians may benefit from asking a more physiologically oriented question: what systemic conditions are driving this metabolic state? In many cases, low T3 levels may reflect broader patterns of energy imbalance, inflammatory signaling, or declining physiologic resilience. These clinical contexts and their associated physiologic interpretations are summarized in Table 1.

**Table 1:** Clinical contexts associated with low T3 states HPT: hypothalamic-pituitary-thyroid; GLP-1: glucagon-like peptide-1; T3: triiodothyronine Reduced circulating T3 may arise in multiple physiologic and pathophysiologic settings. In acute illness and caloric restriction, thyroid hormone alterations often represent adaptive metabolic responses to energetic stress [2,5,32,38]. In contrast, persistent low T3 patterns in chronic inflammatory conditions, cardiometabolic disease, or advanced aging may reflect sustained metabolic suppression and reduced physiologic resilience. Interpretation of non-thyroidal illness syndrome therefore requires careful evaluation of clinical context [19,47,50,51].

Clinical context	Dominant physiologic driver	Typical duration	Clinical interpretation
Acute illness (sepsis, trauma, critical care)	Cytokine-mediated deiodinase shifts, HPT axis suppression	Days to weeks	Adaptive metabolic response
Starvation/caloric restriction	Reduced energy availability and adaptive thermogenesis	Variable	Physiologic energy conservation
Active weight loss/GLP-1 therapy	Sustained caloric deficit and metabolic recalibration	During weight reduction	Adaptive endocrine adjustment
Chronic cardiometabolic disease	Persistent inflammation and metabolic stress	Chronic	Marker of systemic metabolic strain
Advanced aging/frailty	Reduced mitochondrial resilience and muscle loss	Chronic	Potential maladaptive metabolic suppression

Within this perspective, NTIS can be viewed as a metabolic signal rather than a primary endocrine disorder. Interpreting this signal requires the integration of laboratory data with clinical context, disease burden, nutritional status, and functional capacity. Such an approach aligns with the principles of precision endocrinology, which emphasize individualized interpretation of hormonal patterns within the complex physiologic networks that regulate human metabolism.

Future directions

Despite decades of investigation, important questions remain regarding the physiologic significance and clinical implications of NTIS. While substantial evidence supports the adaptive nature of low T3 states during acute illness, far less is known about the long-term consequences of persistent thyroid hormone alterations in chronic disease, aging, and sustained metabolic stress. Addressing these knowledge gaps will require a more nuanced research framework that integrates endocrinology, metabolism, immunology, and aging biology.

One critical priority is the development of longitudinal studies examining the temporal dynamics of NTIS physiology. Most existing research has focused on cross-sectional observations in critically ill patients, limiting insight into how thyroid hormone patterns evolve over time [52]. Prospective studies following patients across acute illness, recovery, and chronic disease progression could help clarify when transient metabolic adaptation resolves and when persistent endocrine alterations emerge. Such work may also identify patient populations in whom low T3 states predict recovery versus those in whom they signal declining physiologic resilience [53].

A second area of investigation involves identifying biomarkers capable of distinguishing adaptive from maladaptive thyroid hormone suppression. Circulating T3 concentrations alone may not adequately reflect tissue-level thyroid hormone signaling. Integration of inflammatory markers, metabolic parameters, and indicators of mitochondrial function may provide a more comprehensive picture of the systemic metabolic state. Emerging approaches, including metabolomics, transcriptomic profiling, and assessment of deiodinase activity, may help elucidate the physiologic context underlying low T3 states [54].

Further research is also needed to better characterize tissue-specific thyroid hormone signaling during illness and metabolic stress. Circulating hormone concentrations represent only one component of thyroid physiology; intracellular T3 availability is largely determined by local deiodinase expression and transport mechanisms [14]. Advances in molecular imaging and tissue-level metabolic analysis may provide new insights into how thyroid hormone signaling is differentially regulated across organs during acute illness, chronic inflammation, and aging.

Finally, the potential clinical implications of persistent low T3 states warrant evaluation through carefully designed prospective trials. While current evidence does not support routine thyroid hormone replacement in acute NTIS, the role of thyroid signaling in chronic metabolic dysfunction remains less clear. Many of the proposed links between NTIS physiology, longevity pathways, metabolic aging, and modern therapeutic interventions remain hypothesis-generating and require prospective validation. Future studies may explore whether interventions targeting underlying drivers, such as inflammation, mitochondrial dysfunction, or nutritional deficits, can restore physiologic thyroid hormone signaling without direct hormone replacement. Such investigations should prioritize patient-centered outcomes, including physical function, metabolic health, and resilience to physiologic stress.

Collectively, these research directions underscore the need to move beyond a static view of NTIS as a transient laboratory phenomenon. Instead, understanding low T3 states will require integrative investigation across multiple physiologic systems and time scales. By clarifying when thyroid hormone downregulation serves protective adaptation and when it reflects emerging metabolic vulnerability, future research may help refine the role of thyroid physiology within the broader landscape of human health, aging, and longevity.

## Conclusions

NTIS has traditionally been framed as a transient and protective endocrine response to systemic illness, an adaptive biochemical pattern that does not reflect intrinsic thyroid disease and generally does not warrant intervention. This interpretation remains appropriate in the setting of acute physiologic stress. However, advances in deiodinase biology, inflammatory signaling, mitochondrial energetics, and aging science suggest that the physiologic meaning of low T3 states is more context-dependent than previously appreciated. Across acute infection, trauma, starvation, and intentional caloric restriction, reduced T3 availability appears to function as a coordinated metabolic downshifting program. By attenuating thermogenesis, anabolic signaling, and mitochondrial oxygen consumption, this endocrine shift conserves energy and reallocates resources toward immune defense and cellular repair. In such settings, NTIS reflects an evolutionarily conserved survival strategy aligned with broader mechanisms of metabolic resilience. Yet when similar biochemical patterns persist in the context of chronic inflammation, cardiometabolic disease, sarcopenia, or advanced aging, the implications may differ. Sustained reductions in thyroid-driven metabolic activity may contribute to impaired mitochondrial function, reduced anabolic capacity, diminished metabolic flexibility, and increased frailty risk. The distinction between adaptive recalibration and maladaptive suppression appears to depend less on hormone concentration alone and more on duration, inflammatory burden, energetic status, and physiologic reserve.

Reframing NTIS through a longevity-oriented and systems-based lens shifts the clinical question from "Should this be treated?" to "What does this metabolic signal represent in this patient?" Such an approach avoids both reflexive intervention and reflexive dismissal. Instead, it encourages contextual interpretation grounded in an understanding of thyroid physiology as a dynamic regulator of energy allocation across the lifespan. Ultimately, low T3 states may represent not a binary disorder but a spectrum of metabolic adaptation. Recognizing when thyroid hormone downregulation reflects protective survival biology, and when it signals declining physiologic resilience, remains a central challenge for precision endocrinology. Importantly, the longevity framework proposed in this review should be viewed as a conceptual and hypothesis-generating model rather than an established clinical paradigm. Future research integrating longitudinal data, tissue-level signaling, and functional outcomes will be essential to clarify this distinction, prospectively validate these concepts, and refine the role of thyroid physiology within modern longevity medicine.
